# Supplementary material for: The structure of a C. neoformans polysaccharide motif recognized by protective antibodies: A combined NMR and MD study
Source: bioRxiv. 2023 Sep 6:2023.09.06.556507. Preprint. [Version 1] doi: 10.1101/2023.09.06.556507 (PMC10508755; doi:10.1101/2023.09.06.556507)
Supplement: Supplement 1 [file media-1.pdf]

Supporting Information for

The structure of a *C. neoformans* polysaccharide motif recognized by protective antibodies: A combined NMR and MD study

Authors:
Audra A. Hargett[a,1], Hugo F. Azurmendi[a,1], Conor J. Crawford[b,c,d], Maggie P. Wear[b], Stefan Oscarson[c], Arturo Casadevall[b], and Darón I. Freedberg[a]

Affiliations:
[a] Laboratory of Bacterial Polysaccharides, Office of Vaccines Research and Review, Center for Biologics Evaluation and Research, U.S. Food and Drug Administration, Silver Spring, MD, USA
[b] W. Harry Feinstone Department of Molecular Microbiology and Immunology, Johns Hopkins Bloomberg School of Public Health, Baltimore, MD, USA
[c]Centre for Synthesis and Chemical Biology, University College Dublin, Belfield, Dublin 4, Ireland
[d] Current address: Department of Biomolecular Systems, Max Planck Institute of Colloids and Interfaces, Potsdam, Germany

This PDF file includes:
Figures S1 to S8
Tables S1 to S4
Supplemental movie legend SM1 to SM3

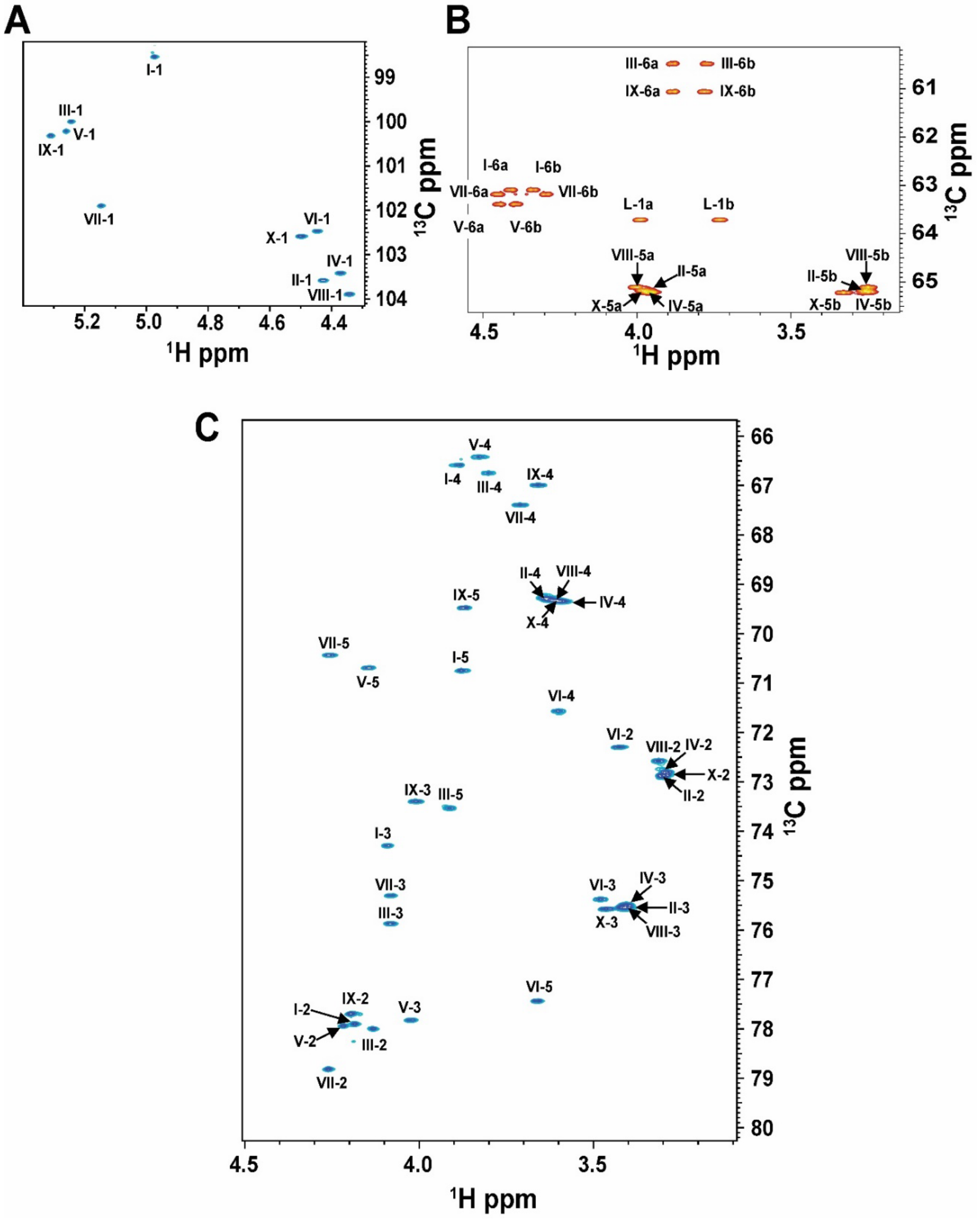

Figure S1: GXM GXM10-Ac$_3$ ^{1}H-^{13}C eHSQC. (A) GXM10-Ac$_3$ Anomeric (^{1}H, ^{13}C) region have a unique chemical shift (^{1}H: 4.3 – 5.4 ppm; ^{13}C: 98-104 ppm) dispersed from other ring ^{1}Hs and ^{13}Cs. **(B)** In the eHSQC experiment, CH_2 resonances are negative, and *O*-acetylated Man C6s (Man[I,V,VII]) are de-shielded from the Man C6s without *O*-acetylation (Man[II, IX]) **(C)** NMR signals for Xyl rings are heavily overlapped, particularly for H2C2, H3C3, H4C4, and H5C5.

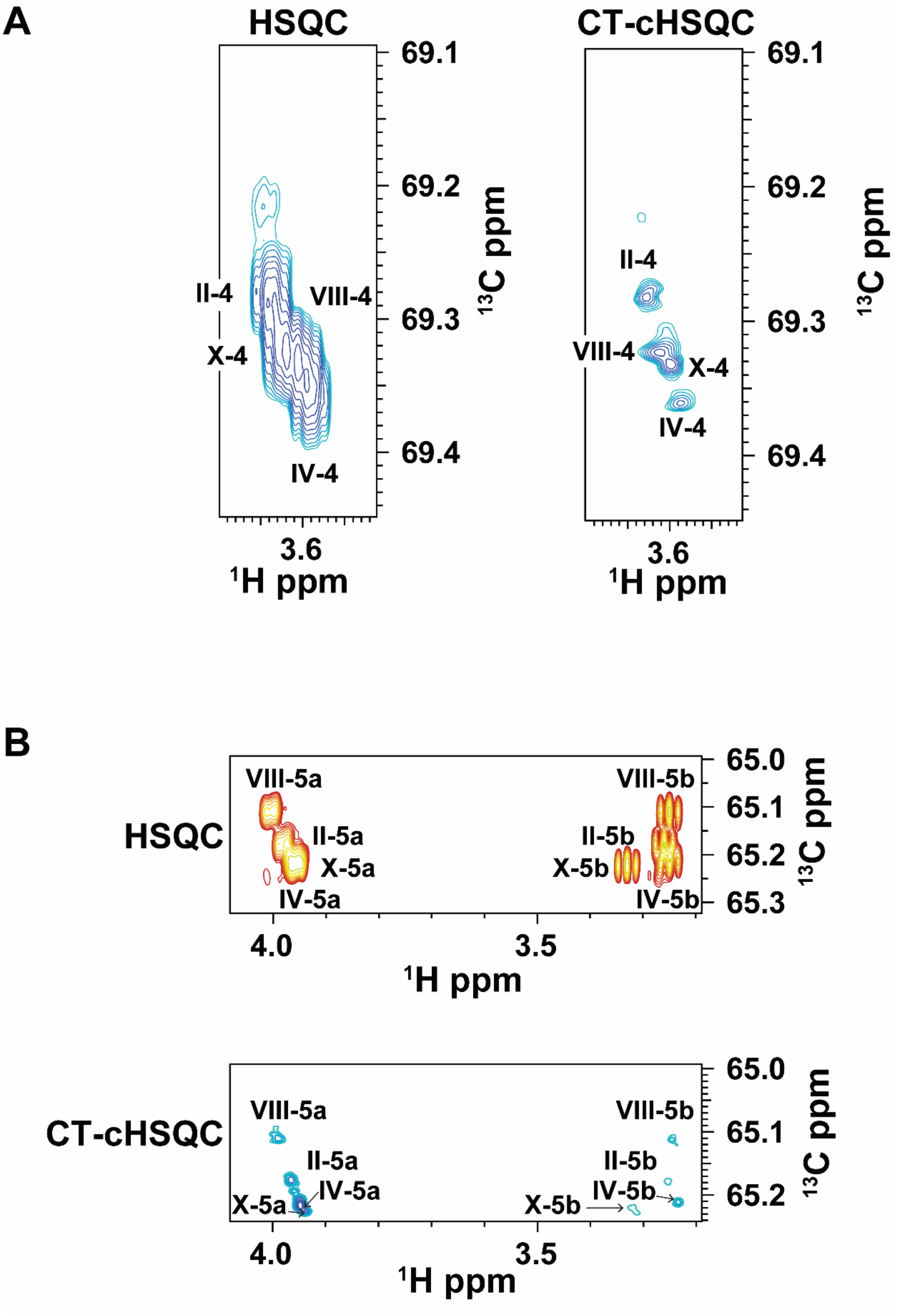

Figure S2: Overlapping H4C4 (A) and H5C5 (B) Xylose resonances in the ^{1}H-^{13}C HSQC experiment are resolved in a ^{1}H-^{13}C CT-cHSQC experiment.

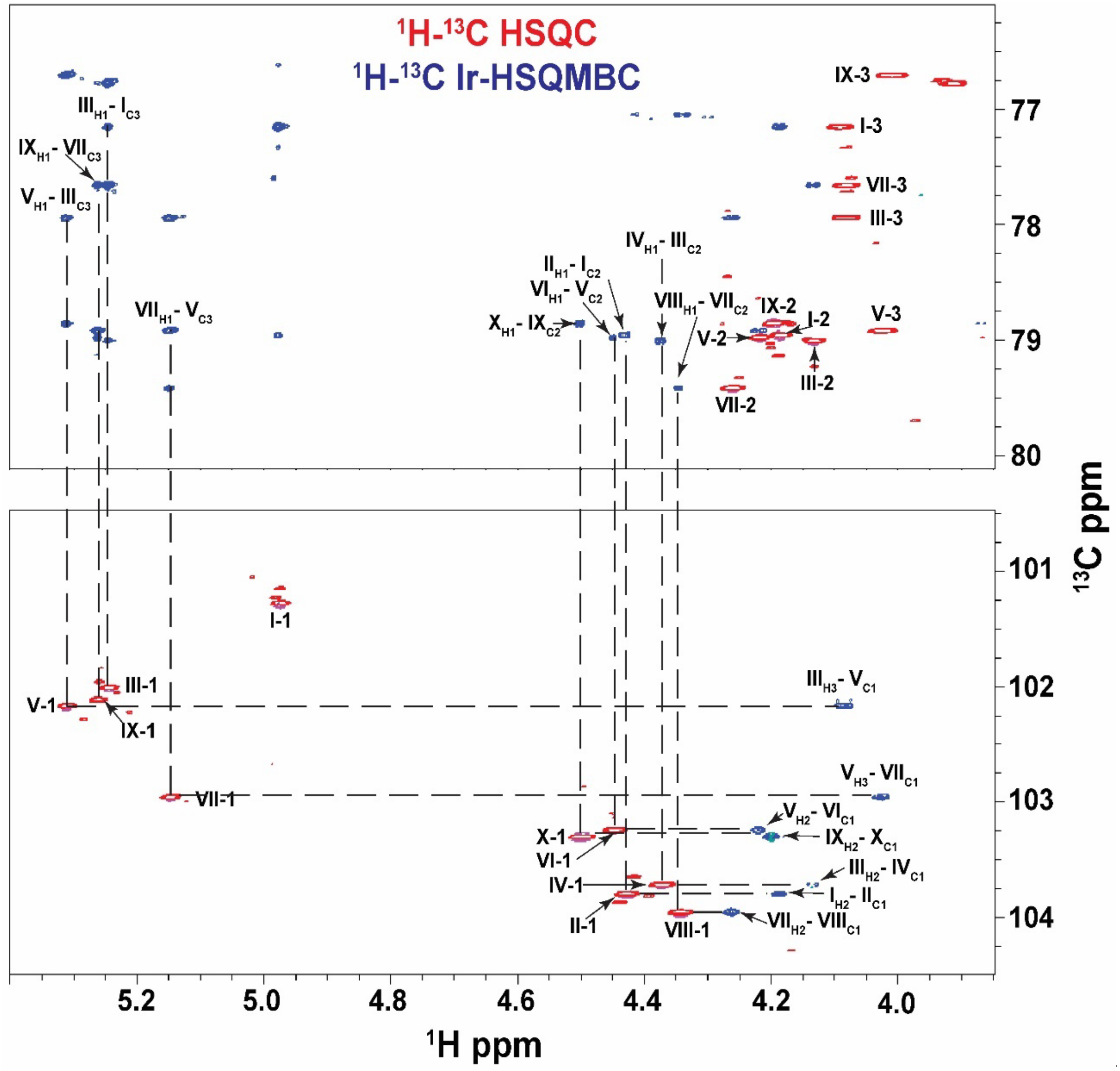

Figure S3: GXM10-Ac$_3$ inter-residue linkages were assigned from inter-glycan crosspeaks obtained in a ^{1}H-^{13}C LR-HSQMBC experiment (blue). ^{1}H-^{13}C HSQC spectrum is overlayed in red.

Table S1: GXM Synthetic Decasaccharide ^{1}H-^{13}C Chemical Shift Assignments

		I	II	III	IV	V	VI	VII	VIII	IX	X
^{1}H	**H-1**	4.97	4.43	5.24	4.37	5.26	4.45	5.15	4.34	5.31	4.50
	**H-2**	4.19	3.31	4.13	3.29	4.22	3.43	4.26	3.32	4.20	3.30
	**H-3**	4.09	3.42	4.08	3.41	4.03	3.48	4.08	3.41	4.01	3.47
	**H-4**	3.89	3.69	3.80	3.60	3.83	3.60	3.71	3.64	3.66	3.63
	**H-5ax**	3.88	3.27	3.91	3.26	4.15	3.66	4.26	3.25	3.87	3.33
	**H-5eq**	-	3.99	-	3.97	-	-	-	4.00	-	3.96
	**H-6a**	4.41	-	3.89	-	4.45	-	4.45	-	3.89	-
	**H-6b**	4.34	-	3.77	-	4.39	-	4.30	-	3.78	-
	**H-CH_3**	2.14	-	-	-	2.20	-	2.17	-	-	-
^{13}C	**C-1**	98.54	103.58	100.00	103.58	100.22	102.48	101.90	103.89	100.32	102.59
	**C-2**	77.91	72.89	78.00	72.89	77.94	72.30	78.82	72.58	77.70	72.87
	**C-3**	74.29	75.53	75.31	75.53	77.83	75.38	75.87	75.55	73.40	75.57
	**C-4**	66.59	69.36	66.75	69.36	66.42	71.57	67.40	69.28	67.00	69.32
	**C-5**	70.75	65.19	73.53	65.19	70.69	77.44	70.44	65.11	69.48	65.22
	**C-6**	63.10	-	60.49	-	65.39		63.19	-	61.06	-
	**C-CO**	174.08	-	-	-	174.16	-	174.12	-	-	-
	**C-CH_3**	20.24	-	-	-	20.58	-	20.57	-	-	-

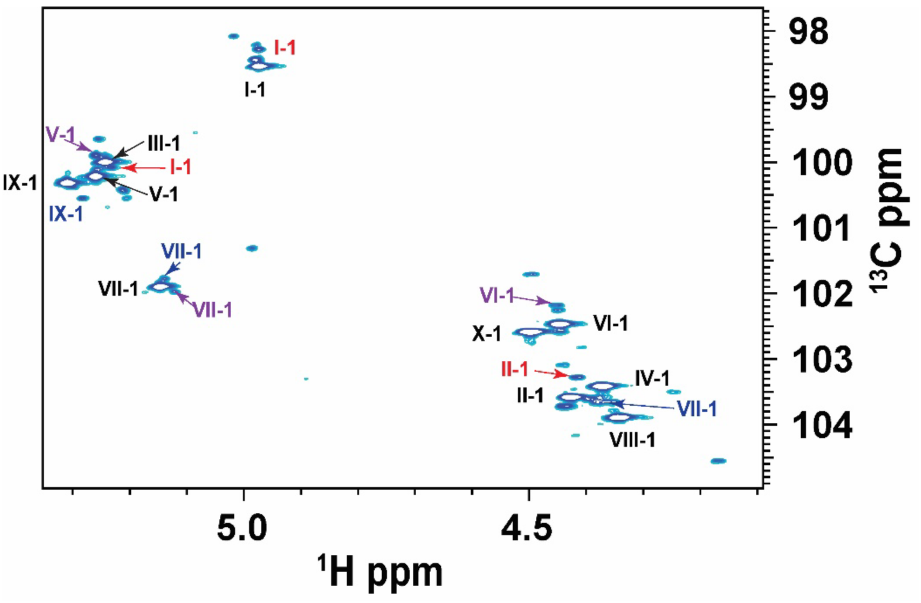

Figure S4: ^{1}H-^{13}C HSQC Anomeric Region showing de-*O*-Acetylated Decasaccharide Resonances. The black labels designate GXM10-Ac3 anomeric signals. The loss of *O*-acetylation at I-Man-C6, V-Man-C6, and VII-Man-C6 are labeled in red, purple, and blue, respectively.

Table S2 ^{1}H-^{13}C Chemical Shift Assignments for de-O-Acetylated GXM Decasaccaharide

		I	II	III	IV	V	VI	VII	VIII	IX	X
**^{1}H**	**H-1**	*4.97*	*4.42*	*5.23*	4.37	5.26*	4.45*	5.12*(5.14)	4.34	(5.28)	4.50
	**H-2**	*4.17*	3.31	4.13	3.29	4.22	3.43	4.26	3.32	4.20	3.30
	**H-3**	*4.08*	3.42	*4.08*	3.41	4.03	3.48	4.08	3.41	4.01	3.47
	**H-4**	*3.85*	3.69	3.80	3.60	3.83	3.60	3.71	3.64	3.66	3.63
	**H-5ax**	3.88	3.27	*3.91*	3.26	4.15	3.66	4.26	3.25	3.87	3.33
	**H-5eq**	-	3.99	-	3.97	-	-	-	4.00	-	3.96
	**H-6a**	4.41	-	3.89	-	4.45	-	4.45	-	3.89	-
	**H-6b**	4.34	-	3.77	-	4.39	-	4.30	-	3.78	-
	**H-CH_3**	2.14	-	-	-	2.20	-	2.17	-	-	-
**^{13}C**	**C-1**	*98.28*	*103.28*	*100.08*	103.58	99.65*	102.18*	101.98*(101.78)	103.89	(100.55)	102.59
	**C-2**	*77.72*	72.89	78.00	72.89	77.94	72.30	78.82	72.58	77.70	72.87
	**C-3**	*74.65*	75.53	*75.41*	75.53	77.83	75.38	75.87	75.55	73.40	75.57
	**C-4**	66.59	69.36	66.75	69.36	66.42	71.57	67.40	69.28	67.00	69.32
	**C-5**	*73.23*	65.19	*73.50*	65.19	70.69	77.44	70.44	65.11	69.48	65.22
	**C-6**	63.10	-	60.49	-	65.39		63.19	-	61.06	-
	**C-CO**	174.08	-	-	-	174.16	-	174.12	-	-	-
	**C-CH_3**	-	-	-	-	-	-	-	-	-	-

Italic denotes chemical shift from the loss of I-Man-C6 O-Acetylation, * denotes the chemcial shift from the loss of V-Man-C6 O-Acetylation, and () denotes loss of VII-Man-C6 O-Acetylation.

Table S3. Measured transglycosidic $^3J_{CH}$ values from ^1H-^{13}C PIP-HSQMBC NMR experiment. All $^3J_{CH}$ have an error of 0.5 Hz. ϕ and ψ torsions were calculated using two different parameterized Karplus relations (see methods). Gold color denotes torsions similar to the average torsions obtained from the MD trajectory.

Tglyc. Bond		$^3J_{CH}$ Exp (Hz)	Calc. Torsion from $J_{meas.}$					
			Tor-S19				Tor-RW	
Man[I]-Man[III]	ϕ	2.5	-123 (+/- 7)	-55 (+/- 8)	40 (+/- 8)	110 (+/- 7)	± 51 (+/- 12)	± 124 (+/- 11)
	ψ	--	--	--	--	--	--	--
Man[III]-Man[V]	ϕ	3.0	-126 (+/- 7)	-51 (+/- 8)	37 (+/- 8)	113 (+/- 6)	± 47 (+/- 11)	± 127 (+/- 10)
	ψ	--	--	--	--	--	--	--
Man[V]-Man[VII]	ϕ	3.5	-130 (+/- 6)	-46 (+/- 8)	32 (+/- 6)	116 (+/- 6)	± 42 (+/- 11)	± 131 (+/- 10)
	ψ	3.4	-129 (+/- 6)	-47 (+/- 8)	33 (+/- 8)	116 (+/- 6)	± 43 (+/- 11)	± 131 (+/- 10)
Man[VII]-Man[IX]	ϕ	2.9	-126 (+/- 7)	-52 (+/- 8)	38 (+/- 8)	112 (+/- 7)	± 48 (+/- 11)	± 126 (+/- 10)
	ψ	4.1	-134 (+/- 6)	-40 (+/- 9)	26 (+/- 9)	121 (+/- 6)	± 36 (+/- 12)	± 136 (+/- 10)
Man[I]-Xyl[II]	ϕ	3.8	-132 (+/- 6)	-44 (+/- 8)	29 (+/- 8)	118 (+/- 6)	± 40 (+/- 11)	± 134 (+/- 10)
	ψ	5.5	-143 (+/- 6)	-25 (+/- 14)	11 (+/- 14)	130 (+/- 6)	± 22 (+/- 17)	± 148 (+/- 11)
Man[III]-Xyl[IV]	ϕ	4.0	-134 (+/- 6)	-41 (+/- 9)	27 (+/- 9)	121 (+/- 6)	± 37 (+/- 12)	± 136 (+/- 10)
	ψ	5.2	-142 (+/- 6)	-28 (+/- 16)	14 (+/- 16)	128 (+/- 6)	± 25 (+/- 19)	± 146 (+/- 11)
Man[V]-GlcA[VI]	ϕ	4.1	-134 (+/- 6)	-41 (+/- 9)	27 (+/- 9)	121 (+/- 6)	± 37 (+/- 12)	± 136 (+/- 10)
	ψ	4.9	-139 (+/- 6)	-33 (+/- 11)	19 (+/- 11)	126 (+/- 6)	± 29 (+/- 14)	± 143 (+/- 5)
Man[VII]-Xyl[VIII]	ϕ	4.2	-135 (+/- 6)	-40 (+/- 9)	25 (+/- 9)	121 (+/- 6)	± 36 (+/- 12)	± 137 (+/- 10)
	ψ	4.8	-139 (+/- 6)	-34 (+/- 10)	20 (+/- 10)	125 (+/- 6)	± 30 (+/- 13)	± 142 (+/- 10)
Man[IX]-Xyl[X]	ϕ	3.5	-130 (+/- 6)	-46 (+/- 8)	32 (+/- 8)	117 (+/- 6)	± 42 (+/- 11)	± 131 (+/- 10)
	ψ	4.8	-140 (+/- 6)	-33 (+/- 10)	19 (+/- 10)	125 (+/- 6)	± 29 (+/- 14)	± 142 (+/- 10)

Table S4. Average ϕ and ψ torsions from MD trajectory and predicted $^3J_{CH}$ values. Gray denotes calculated $^3J_{CH}$ values that do not agree well with experimental torsions.

Tglyc. Bond		MD Tor.	$^3J_{CH}$ Calc. from MD	
			J-S19	*J*-RW
Man[I]-Man[III]	ϕ	**-53** (+/- 12)	**2.7** (+/-1.1)	**2.0** (+/-1.0)
	ψ	**-8** (+/- 21)	*5.3* (+/-0.9)	*4.3* (+/-1.0)
Man[III]-Man[V]	ϕ	**-51** (+/- 14)	**2.9** (+/-1.1)	**2.2** (+/-1.0)
	ψ	**-5** (+/- 22)	*5.3* (+/-1.0)	*4.3* (+/-1.0)
Man[V]-Man[VII]	ϕ	**-45** (+/- 15)	**3.4** (+/-1.1)	**2.6** (+/-1.0)
	ψ	**5** (+/- 24)	*4.9* (+/-1.3)	*4.2* (+/-1.1)
Man[VII]-Man[IX]	ϕ	**-47** (+/- 14)	**3.3** (+/-1.1)	**2.5** (+/-1.0)
	ψ	**6** (+/- 22)	*5.0* (+/-1.2)	*4.3* (+/-1.0)
Man[I]-Xyl[II]	ϕ	**41** (+/- 20)	**2.4** (+/-1.5)	**2.8** (+/-1.3)
	ψ	**14** (+/- 25)	**4.7** (+/-1.7)	**4.0** (+/-1.3)
Man[III]-Xyl[IV]	ϕ	**41** (+/- 31)	**2.6** (+/-1.8)	**2.9** (+/-1.3)
	ψ	**17** (+/- 26)	**4.3** (+/-1.8)	**3.8** (+/-1.4)
Man[V]-GlcA[VI]	ϕ	**39** (+/- 30)	**2.7** (+/-1.9)	**3.0** (+/-1.4)
	ψ	**32** (+/- 33)	**2.9** (+/-1.9)	**2.8** (+/-1.5)
Man[VII]-Xyl[VIII]	ϕ	**40** (+/- 23)	**2.6** (+/-1.6)	**3.0** (+/-1.3)
	ψ	**30** (+/- 27)	**3.4** (+/-2.0)	**3.2** (+/-1.5)
Man[IX]-Xyl[X]	ϕ	**40** (+/- 22)	**2.4** (+/-1.4)	**2.8** (+/-1.2)
	ψ	**20** (+/- 30)	**4.0** (+/-1.8)	**3.5** (+/-1.3)

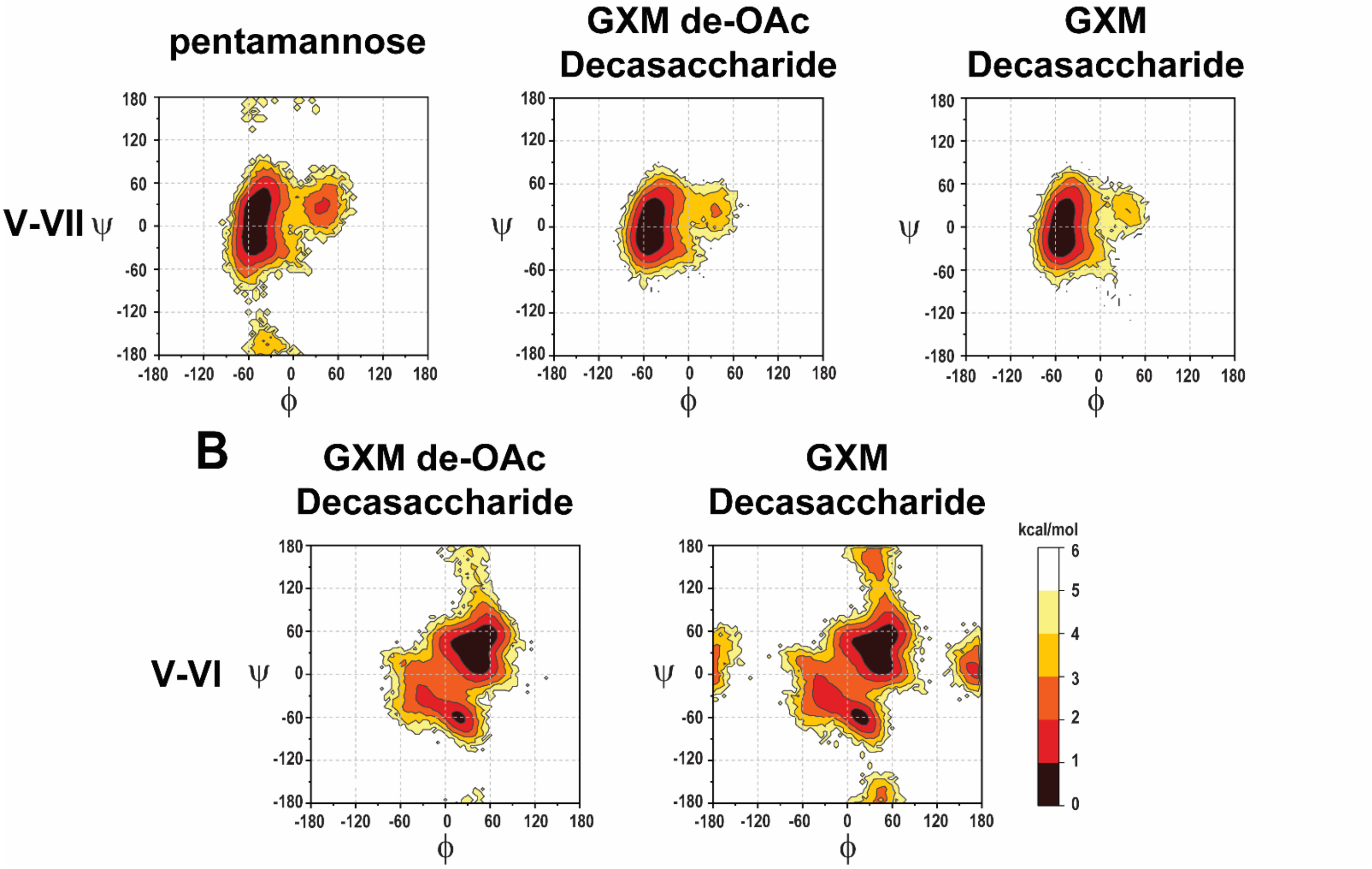

Figure S5: Effect of Substitution on GXM10-Ac$_3$ Structure. A (ϕ, ψ) transglycosidic torsion angle population analysis of a pentamannose, GXM10, and GXM10-Ac$_3$ were compared. **(A)** The Man[V]-Man[VII] transglycosidic torsion heatmap suggests a decrease in flexibility upon substitution of Xyl and *O*-acetylation. **(B)** *O*-acetylaton does not seem to have an effect on the torsional landscape of the Man-Xyl/GlcA linkages.

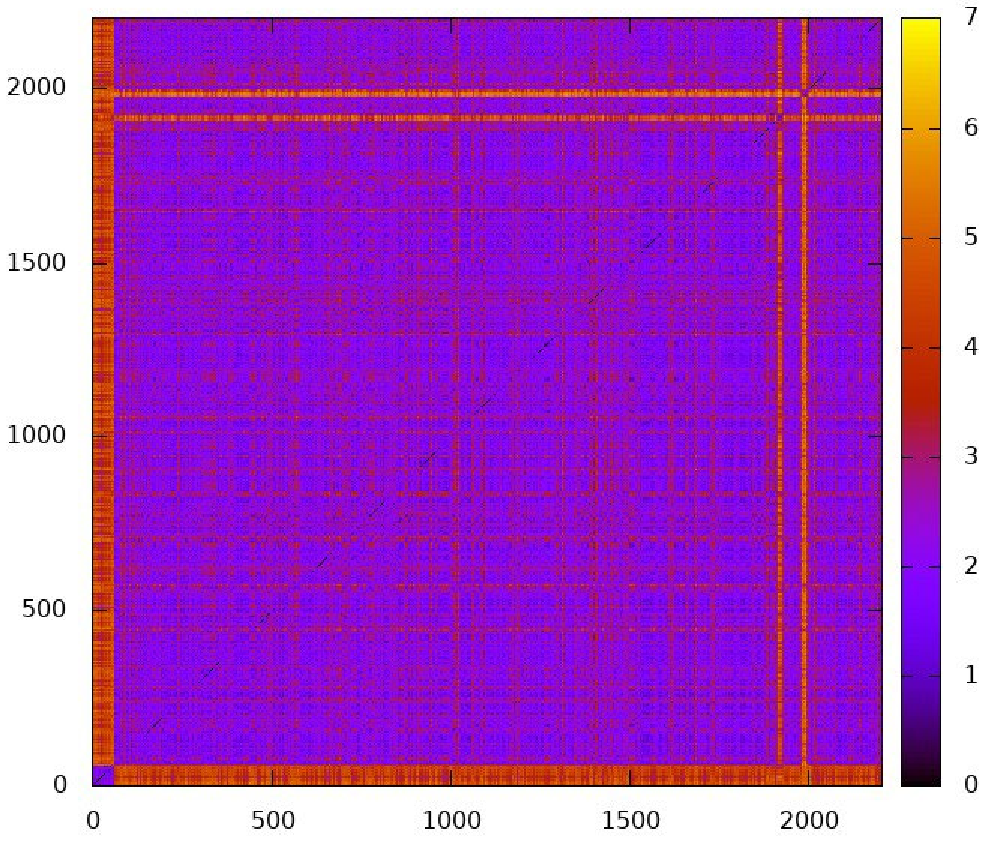

.

Figure S6. 2D-RMSD map of GXM10-Ac_3, analyzing 2200 frames over 2.2 μs MD. RMSD calculated using the 6-ring atoms for all 10 glycan residues in GXM10-Ac_3 (total of 60 atoms). The first 75 ns correspond to a high energy conformation that is not revisited once a steady state is reached. Based on this, analysis of the GXM10-Ac_3 MD was limited the resulting trajectory starting at 100 ns up to 2100 ns (2 μs in total)

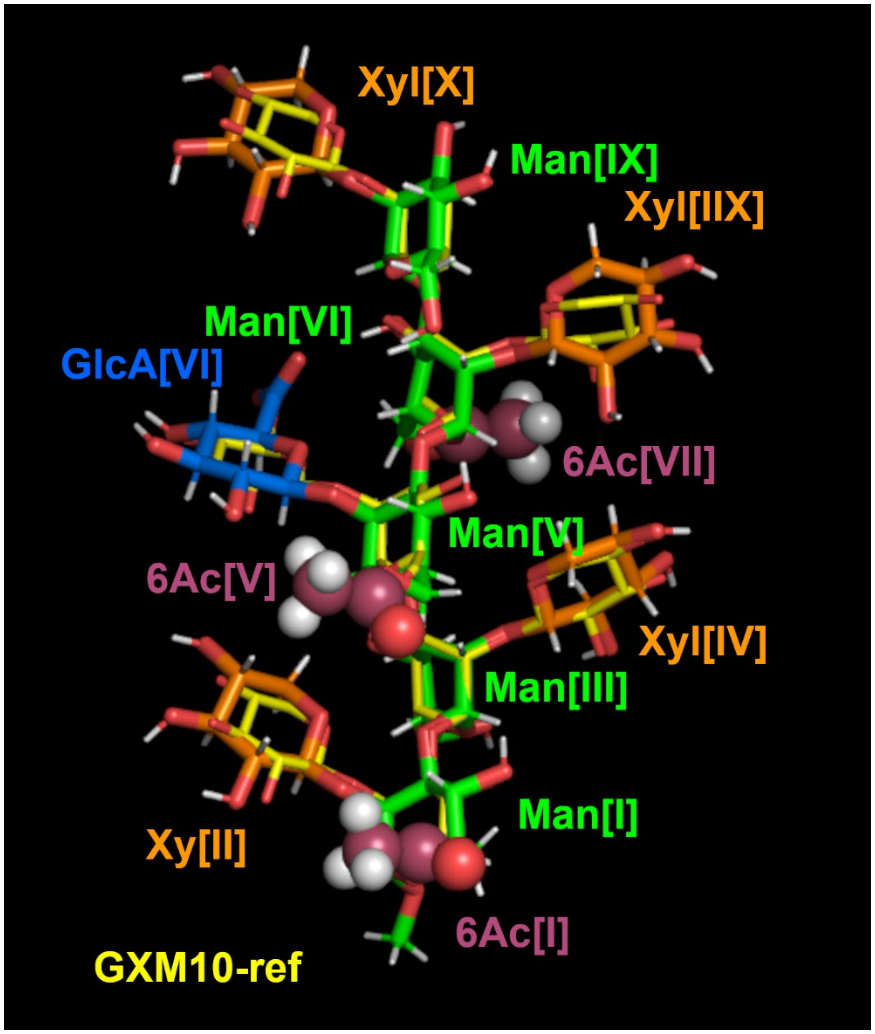

Figure S7. Overlay of GXM10ref (yellow) and the GXM10-Ac_3 model with lowest RMSD to GXM10ref (RMSD = 0.56 Å, 60 ring atoms) from the 2 μs MD trajectory.

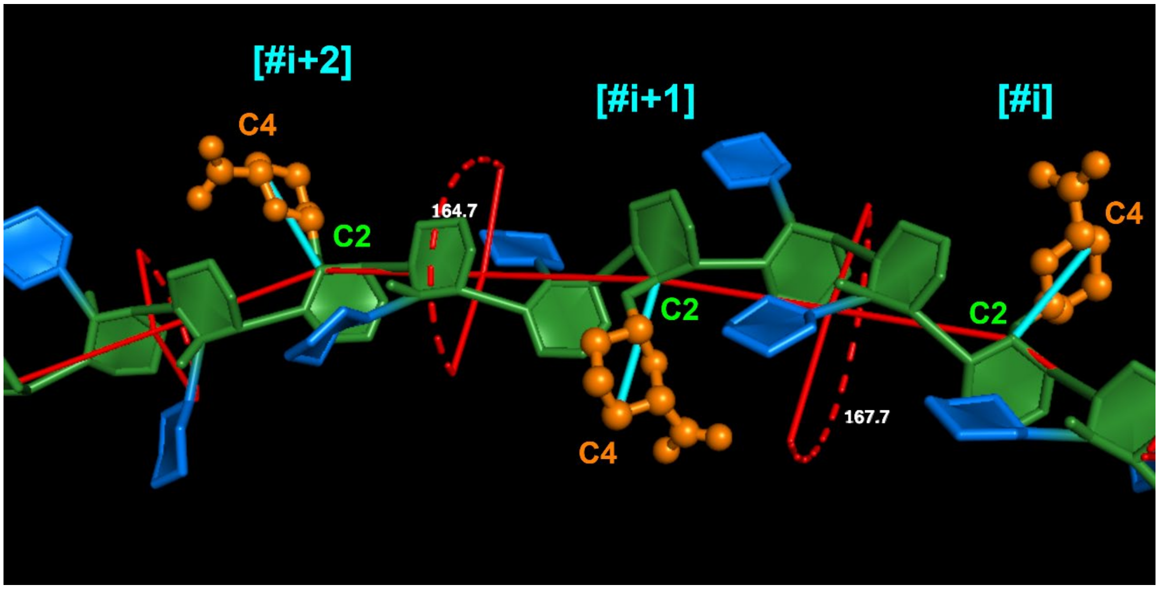

Figure S8. Vector definitions to calculate the twist pitch of the GXM PS. Vectors between atoms Man{GlcA}-C2 and GlcA-C4 were defined for each repeating unit (RU) [cyan lines], then dihedral angles between consecutive vectors were calculated throughout the trajectory. The figure shows a portion of the final frame of GXM12RU to illustrate the general procedure employed to determine inter-RU's dihedrals to generate Figure 4C.

Movie S1. GXM10-ref shown in Figure 3A rolled around the *y*-axis to better appreciated its 3D structure.

Movie S2. The trajectory of the linked Man[V]6Ac-GlcA shown in Figure 3B, to better illustrate the branch dynamics. The trajectory was ‘smoothed’ to reduce high frequency movements.

Movie S3. Last 500 ns of the MD trajectory of GXM12RU. Frames were RMSD minimized to the ring atoms of the colored residues. The trajectory was ‘smoothed’ to reduce high frequency movements.
